# The Potential of Co-Fermentation of Whole-Plant Cassava with *Piper sarmentosum*: A Comprehensive Study of Fermentation Quality, Antioxidant Activity, Bacterial Community Structure, and Microbial Ecological Networks in Novel Foods

**DOI:** 10.3390/foods13132126

**Published:** 2024-07-03

**Authors:** Mao Li, Renlong Lv, Wenjun Ou, Songbi Chen, Hanlin Zhou, Guanyu Hou, Xuejuan Zi

**Affiliations:** 1 Key Laboratory of Ministry of Education for Genetics and Germplasm Innovation of Tropical Special Trees and Ornamental Plants, Key Laboratory of Germplasm Resources of Tropical Special Ornamental Plants of Hainan Province, School of Tropical Agriculture and Forestry, Hainan University, Danzhou 571737, China; limao@catas.cn; 2 Tropical Crops Genetic Resources Institute, Chinese Academy of Tropical Agricultural Sciences, Danzhou 571737, China; lvrenlong@catas.cn (R.L.); wenjunou@catas.cn (W.O.); songbichen@catas.cn (S.C.); zhouhanlin@catas.cn (H.Z.); guanyuhou@catas.cn (G.H.)

**Keywords:** co-ferment, whole-plant cassava, *Piper sarmentosum*, antioxidant activity, bacterial co-occurrence network

## Abstract

The objective of this study was to explore the preservation of food products through the co-fermentation of whole-plant cassava and *Piper sarmentosum* (PS) without additives. We assessed fermentation quality, antioxidant activity, bacterial community structure, function profile, and microbial ecological network features. Our results demonstrate that co-fermentation of whole-plant cassava with 10% PS significantly improves food quality. The co-fermented samples exhibited enhanced lactic acid concentrations and increased antioxidant activity, with reduced pH values and concentrations of acetic acid, butyric acid, and ammonia-N(NH_3_-N) compared to whole-plant cassava fermented alone. In addition, PS addition also optimized microbial community structure by elevating the total abundance of lactic acid bacteria and influenced bacterial predicted functions. Furthermore, our analysis of co-occurrence networks reveals that co-fermentation impacts microbial network features, including module numbers and bacterial relative abundances, leading to altered complexity and stability of the networks. Moreover, out study also highlights the impact of ferment undesirable bacteria like *Pseudomonas aeruginosa* and unclassified_*Muribaculaceae* playing crucial roles in microbial network complexity and stability. These findings provide valuable insights into the anaerobic fermentation process and offers strategies for regulating food fermentation quality.

## 1. Introduction

Cassava (*Manihot esculenta* Crantz) is a widely cultivated crop in tropical regions and ranks as the sixth largest food crop globally, providing sustenance to around 600 million people worldwide [1]. Each part within the cassava plant is dense in nutrients, making them suitable for human and animal consumption. Cassava leaves are rich in essential nutrients such as protein, minerals, and active components [2,3,4]; cassava stems contain moderate dietary fibers and starch [5,6]; and cassava tubers are abundant in starch and carbohydrates [7,8,9,10]. Considering these nutritional qualities, cassava plants are a rich source of essential nutrients and serve as a vital food source in Africa, Latin America, and Southeast Asia [7,8,9,10]. This study innovatively combines various parts of cassava as a comprehensive utilization as “whole-plant cassava”, resulting in a more balanced nutritional composition and a healthier food. However, due to the seasonal limitations of cassava, it is crucial to determine a process to preserve the product long-term. One viable option of the whole-plant cassava utilization is anaerobic fermentation.

Anaerobic fermentation holds significant importance in the preparation and preservation of plant products. It not only extends the shelf life of crop plants, including cassava, but also enhances their digestibility, improves organoleptic and nutritional properties, reduced toxic substances, stabilizes product texture, and facilitates the creation of value-added and innovative bioproducts [11,12]. The quality of fermentation depends on various factors, including the fermentation methods (inoculated or spontaneous fermentation) and fermentation additives (microorganisms, organic acid, alkaline and alcohol, etc.). Failed fermentation can lead to shortened shelf life, waste of raw materials, an increase in pathogenic bacteria, endangering food safety, and causing economic losses [11,13,14]. Despite the importance of this preservation method, there is a lack of information regarding the fermentation characteristics and modulation technology for whole-plant cassava, necessitating further research in this area.

In recent years, natural functional plants with antibacterial and antioxidant properties have been used to improve the quality of food fermentation [14]. These plants contained bio-active components such as flavonoids, phenolic compounds, polysaccharide, and essential oils, which increases flavor and active ingredient content while improving the antioxidant activity of food [14,15,16]. *Piper sarmentosum* Roxb. (PS), a plant with significant medicinal and edible value, has strong antibacterial and antioxidant effects due to its alkaloids and phenols [17,18,19]. Meanwhile, PS has not yet been applied in food anaerobic fermentation, and its effectiveness is also unknown. Therefore, co-fermentation of PS and whole-plant cassava is hypothesized to enhance fermentation quality, antioxidant activity, and preserve nutritional components.

Food anaerobic fermentation is a complex microbial ecology process characterized by microbial co-occurrence networks, network modules, and network stability [20,21]. Our previous studies have shown that fermentation quality and microbial co-occurrence networks are interrelated, and two materials co-fermented altered the microbial network features [22]. Meanwhile, few factors such as epiphytic microbiota, lactic acid bacteria (LAB) inoculants, storage temperatures, and fermentative time can influence microbial co-occurrence networks and modules, thereby enhancing fermentation quality [23,24,25]. Analyzing these microbial ecological network characteristics is essential for understanding the fermentation process and precisely regulate ferment whole-plant cassava food quality.

Given the information presented, we hypothesize that whole-plant cassava co-fermented with PS can enhance the fermentation quality and antioxidant activity, leading to the production of functional food. Notably, no prior studies have focused on the intricate interplay of the bacterial community, functional attributes, and microbial ecological network characteristics in co-fermented whole-plant cassava and medicinal plant. Consequently, this study aims to explore the most efficient and superior method for fermenting whole-plant cassava and PS in varying ratios. Our focus lies on evaluating not only fermentation quality and antioxidant activity but also delving into the intricacies of bacterial community structure, function profiles, microbial network characteristics, network complexity, and stability. We hope this study could provide valuable insights into the ferment microbial community and microecological network, and sheds light on the microbial mechanisms underpinning the production of high-quality functional foods through co-fermentation with medicinal plants. Ultimately, our aim is to pinpoint the optimal ratio of PS addition, paving the way for a refined and effective production process.

## 2. Materials and Methods

### 2.1. Sample Preparation

The study was conducted at the Chinese Academy of Tropical Agricultural Sciences (CATAS) in Danzhou, Hainan, China, located at a longitude of 109°30′ E, a latitude of 19°30′ N, and an altitude of 149 m. Whole-plant cassava (SC14) was planted in experimental field of CATAS on 15 March 2021, and harvested on 15 November 2021. On the same day, *Piper sarmentosum* Roxb. (PS) was collected from three randomly chosen plots in experimental field of CATAS. Then, the chopped whole-plant cassava and PS were grouped into small piles.

The freshly harvested materials were finely chopped into approximately 2-centimeter-sized pieces using a high-quality grass chopper (9Z-2.5; Jinhongxing Industrial Company, Zhengzhou, China). The chopped whole-plant cassava and PS were grouped into small piles. Then the chopped whole-plant cassava and PS were randomly sampled from their respective small piles and evenly mixed in four different ratios: 100% whole-plant cassava without PS (CFCK); 90% whole-plant cassava with 10% PS (CF10PS); 80% whole-plant cassava with 20% PS (CF20PS); and 70% whole-plant cassava with 30% PS (CF30PS). Each blend, weighing 500 g, was carefully homogenized and vacuum-sealed in sterile plastic bags measuring 30 × 10 × 4 cm. In total, 12 bags, comprising four mixed ratios with three replicates each, were prepared and then incubated at room temperature. Samples from these mixed ferments were collected on day 60 for comprehensive analysis, including assessments of chemical composition, antioxidant activity, fermentation quality, and microbial community dynamics. The detailed process of the above method was followed according to the protocol described by Li et al. [22].

### 2.2. Antioxidant Activity, Culture-Based Microbial, and Fermentation Index Analysis

The fermentation samples were subjected to a heat treatment at 65 °C for 72 h to determine the dry matter (DM) content. Subsequently, the dried materials were ground and sieved through a 1 mm screen for the analysis of water-soluble carbohydrates (WSC), crude protein (CP), neutral detergent fiber (NDF), and acid detergent fiber (ADF). These parameters were measured following the protocols outlined by the Association of Official Analytical Chemists [26]. The starch content was determined utilizing a total starch determination kit, following the experimental procedures described by Bai et al. [27]. To assess the antioxidant capacity, methods outlined by Li et al. [28] were employed. The lactic acid bacteria (LAB), molds, and coliform bacteria quantities of fresh samples before ensiling were counted using by De Man–Rogosa–Sharpe agar, potato dextrose agar, and violet red bile agar, respectively [29]. Fifty grams of ferment was blended with 200 mL of distilled water, followed by incubation at 4 °C for 24 h and then half of them filtered by millipore filter (0.45 um). Half of each extract sample without filtration was stored at −80 °C for microbial diversity analysis, while the remaining portion was used for the analysis of fermentation quality [28]. The pH was measured using a glass electrode pH meter. Lactic acid, acetic acid, propionic acid, and butyric acid concentrations were determined using high-performance liquid chromatography (HPLC, SHIMADZU-10A, Kyoto, Japan; Column, C18, SHIMADZU-10A, Kyoto, Japan; Eluent: 1 mL·min^−1^; detector: 210 nm; volume: 10 μL; temperature: 35 °C) following the assay protocol described by Liu et al. [30]. The detailed process of the above analysis was improved and optimized according to the protocol described by Li et al. [22].

### 2.3. Microbial Community and Function Profile Analysis

The aforementioned extracts without filtration were used for microbial community analysis. Bacterial DNA was extracted using the TGuide S96 Magnetic Soil/Stool DNA Kit (Tiangen Biotech (Beijing) Co., Ltd., Beijing, China). DNA concentration of each extract sample was quantified using the Qubit dsDNA HS Assay Kit and Qubit 4.0 Fluorometer (Invitrogen, Thermo Fisher Scientific, Waltham, MA, USA). PCR amplification was performed following the protocols outlined by Bai et al. [23]. The full-length 16S rRNA gene was amplified from the genomic DNA of each sample using the universal primer set 27F: AGRGTTTGATYNTGGCTCAG and 1492R: TASGGHTACCTTGTTASGACTT. Products from the PCR assay were then sequenced on a PacBio Sequel platform (Pacific Biosciences, Menlo Park, CA, USA) according to the standard procedures of Biomarker Technologies (Beijing, China).

The raw reads generated from sequencing were filtered and demultiplexed using SMRT Link software (version 8.0) with parameters set at minPasses ≥ 5 and minPredictedAccuracy ≥ 0.9, resulting in circular consensus sequencing (CCS) reads. The lima (version 1.7.0) was employed to assign CCS sequences to the respective samples based on their barcodes. CCS reads without primers and those falling outside the length range (1200–1650 bp) were removed through recognition of forward and reverse primers and quality filtering using the Cutadapt quality control process (version 2.7). The UCHIME algorithm (v8.1) was utilized for detecting and eliminating chimera sequences, to produce clean reads. Sequences with a similarity of ≥97% were clustered into operational taxonomic units (OTU) using USEARCH (v10.0), and OTUs with abundances < 0.005% were removed.

Taxonomic annotation of the ferment bacterial OTUs was performed using the naive Bayes classifier in QIIME2 with the SILVA database (release 138) at a similarity cut-off of 70% [31]. Alpha diversity (represented by OTU number and Shannon/Simpson’s diversity indices) was calculated and displayed using QIIME2(2023.1) and R (v3.1.1) software, respectively. Beta diversity, evaluated via non-metric multidimensional scaling (NMDS), assessed the similarity between microbial communities from different extract samples, and significant taxonomic differences among groups were tested using linear discriminant analysis (LDA) effect size (LEfSe) [32]. Bacterial functions were predicted from the Kyoto Encyclopedia of Genes and Genomes (KEGG) database using Phylogenetic Investigation of Communities by Reconstruction of Unobserved States 2 (PICRUSt2, v2.3.0_b), which predicts functional abundance based on marker gene sequences in the sample [33]. Microbial network analysis, encompassing co-occurrence networks, network modules, complexity, and stability, were conducted using GGCLUSTERNET [21] available on GitHub (https://github.com/taowenmicro/ggClusterNet, accessed on 10 May 2023). To explore the impact of microbial community composition on the microbial networks, correlation heatmaps and canonical correlation analysis (CCA) between network properties and microbial communities were performed. All data were analyzed using the BMKCloud Platform (v1.0) (www.biocloud.net). The detailed process of the above analysis was improved and optimized according to the protocol described by Li et al. [22].

The sequencing data were deposited in the Sequence Read Archive (SRA) with the accession number of PRJNA1027994.

### 2.4. Statistical Analysis

All results are reported as the mean of three replicate groups. The data pertaining to fermentation quality, chemical composition, and antioxidant activity data for each treatment were subjected to one-way analysis of variance (ANOVA). All statistical analyses were performed using SPSS 20 and GraphPad Prism 8.0. Orthogonal polynomial contrasts, specifically linear correlation, were used to evaluate the impact of different ratios of whole-plant cassava and PS mixtures. Duncan’s multiple-range method was utilized for pairwise comparisons of means, and significance was determined at a threshold of *p* < 0.05.

## 3. Results and Discussion

### 3.1. Characteristics of Fresh Whole-Plant Cassava and PS

Table 1 presents the chemical composition and epiphytic microbial counts of fresh whole-plant cassava and PS. The whole-plant cassava contained 312.5 g/kg dry matter (DM) per 1 kg of fresh matter (FM) and was rich in protein (158.6 g/kg DM) and carbohydrates (177.2 g/kg DM WSC, 215.9 g/kg DM starch), and high in fiber (116.8 g/kg DM ADF, 158.9 g/kg DM NDF). In contrast, PS had a lower dry matter content (192.4 g/kg FM), lower concentrations of protein (41.9 g/kg DM) and carbohydrates (38.7 g/kg DM WSC, 12.4 g/kg DM starch), and fiber content (62.5 g/kg DM ADF, 86.1 g/kg DM NDF). All of the chemical compositions shown significant differences between two raw materials (*p* < 0.05). Despite similar counts of epiphytic lactic acid bacteria (*p* > 0.05) in both cassava and PS, whole-plant cassava had higher mold and *Enterobacter* counts (*p* < 0.05).

From our results, whole-plant cassava demonstrates a more balanced nutrient profile compared to its leaves, stem, and tuber, containing abundant protein, water-soluble carbohydrates, and starch while having lower fiber content [2,6,10]. However, the nutritional value of fermented whole-plant cassava is still not well understood. Previous research has indicated that PS is rich in nutrients such as amino acids and minerals, as well as active compounds like alkaloids and phenolics [34]. These nutrients have antibacterial and antioxidant properties, which make them valuable for both medicinal and edible purposes [17,18]. Therefore, co-fermenting whole-plant cassava and PS may be advantageous in inhibiting harmful bacteria and preserving nutrients. Additionally, the presence of more epiphytic lactic acid bacteria and fewer undesirable bacteria in both whole-plant cassava and PS suggests that their co-fermentation has the potential to produce high-quality food products.

### 3.2. Nutrition Composition, Fermentation Characteristics, and Antioxidant Capacity of Co-Fermented Whole-Plant Cassava and PS

Nutrition composition, fermentation characteristics, and antioxidant capacity of co-fermented whole-plant cassava and PS are presented in Table 2. The proportions of PS in the co-fermented whole-plant cassava impact the nutrition composition significantly. The CFCK treatment, with the highest content of dry matter (DM), crude protein (CP), acid detergent fiber (ADF), neutral detergent fiber (NDF), water-soluble carbohydrates (WSC), and starch (*p* < 0.05) shows a clear decrease as the PS proportion increases. Both treatment and linear proportion of PS have a significant effect on the nutritional composition of the co-fermented samples (*p* < 0.05).

Anaerobic fermentation is a promising technology for preserving cassava products. This method offers a multitude of advantages, including the degradation of toxic substances, enhancement of flavor, preservation of nutrients, cost reduction, and decreased water consumption [11]. Supporting this, a study conducted Kouassi et al. [35] highlighted the effectiveness of anaerobic fermentation in preserving nutrients, with the quality of the fermented food influenced by geographical factors. In our research, both whole-plant cassava and PS were found to be rich sources of nutrients. Post-fermentation, there was a notable preservation of nutrient composition, especially concerning crucial elements such as CP, WSC, and starch. The ammonia-N content serves as an indicator of protein or amino acids decomposition [36]. Significantly lower ammonia-N content in our co-fermentation treatments suggest a higher retention of crude protein [37]. Additionally, there was a substantial reduction in WSC and starch across all treatments, indicating that these fermentation substrates were effectively converted by lactic acid bacteria (LAB) into high-yield organic acids. Similar findings were reported by Zeng et al. [29] and Wang et al. [24] in their studies on co-fermentation involving materials with diverse nutritional profiles. It is crucial to note that the changes in the nutritional composition during co-fermentation primarily stem from alterations in the mixture ratio of the two raw materials. A more balanced nutrient composition was found to be particularly conducive to silage fermentation, emphasizing the importance of optimizing the raw material proportions for achieving better fermentation outcomes.

There is a discernible difference in fermentation quality between co-fermented whole-plant cassava and singly fermented whole-plant cassava, as evident in Table 2. As the proportion of PS increases, the pH value decreases while lactic acid content rises (except CF20PS) (*p* < 0.05). Additionally, the CF20PS treatment exhibited the highest acetic acid concentration, whereas CF10PS and CF30PS treatments had lower levels (*p* < 0.05). Conversely, propionic acid, butyric acid, and NH3-N were most abundant in the CFCK treatment and significantly reduced in PS-containing treatment s (*p* < 0.05). Overall, incorporating the lowest proportion of PS effectively reduced pH, acetic acid, propionic acid, butyric acid, and ammonium nitrogen content while significantly increasing lactate content, thereby correspondingly enhancing fermentation quality.

pH value serves as a pivotal parameter for evaluating fermentation quality. In our study, whole-plant cassava fermented alone displayed the highest pH value, which significantly decreased in the mixed silage group. This aligns with the findings of Kouassi et al. [35], who observed well-maintained pH levels in fermented cassava products in Côte d’Ivoire. Moreover, our fermented whole-plant cassava exhibited notably lower pH values compared to fermented cassava foliage in our previous research, indicating that whole-plant cassava is a better silage material [38]. The pH values in the mixed fermented samples were close to or lower than 4.2, the threshold for well-preserved silage. Similar trends were reported by Wang et al. [39] and Zeng et al. [29] in their studies on mixed fermented samples. Additionally, the lactic acid (LA) content in all three fermented treatments were relatively high (except CF20PS). This suggests that the low pH fermentation environment promotes homofermentation, leading to the production of a significant amount of lactic acid. However, the CF20PS treatment exhibited the highest pH and acetic acid content, indicating that heterofermentation impeded the improvement in fermentation quality. This phenomenon resonates with the findings of Guo et al. [40] and Wang et al. [39].

Furthermore, we observed elevated levels of butyric acid and ammonia-N in the CFCK treatment, a trend consistent with the variation in pH values. Typically, higher pH values are conducive to the growth of undesirable bacteria [24], leading to the production of butyric acid and ammonia-N, both harmful to the preservation of food nutrients. The rich nutrient content in whole-plant cassava may further facilitate the growth of undesirable bacteria, and contribute to higher butyric acid and ammonia-N content. In contrast, PS contains potent antibacterial and antioxidant compounds that inhibit undesirable bacteria, while promoting the growth of LAB. This promotes lactic acid fermentation and improves overall fermentation quality. Previous studies have highlighted the efficacy of functional herbs in enhancing fermentation quality by reducing butyric acid and ammonium nitrogen content [15,16,41]. In summary, the co-fermentation of whole-plant cassava and PS achieved outstanding fermentation quality. This result provides new ideas for food processing in tropical regions, especially for staple food with high starch or sugars that are difficult to preserve for a long time. PS can emerges as a promising fermented additive for this type of food, which is of great significance in preserving the nutritional components and extending its shelf life.

Comparing the fermentation of whole-plant cassava alone with co-fermentation, the co-fermentation whole-plant cassava treatments exhibited significantly higher concentrations of total antioxidant capacity (T-AOC), superoxide dismutase (SOD), and glutathione peroxidase (GSH-Px) (*p* < 0.05), while the catalase (CAT) concentration was highest in the CFCK treatment (*p* < 0.05). Interestingly, these antioxidant indexes did not significantly differ among the three co-fermentation treatments (*p* > 0.05). Previous research has indicated that anaerobic fermentation enhances the concentration of T-AOC, SOD, and GSH-Px, while reducing CAT levels, thereby enhancing antioxidant ability, which aligns with our findings [28,42]. Moreover, we observed that the antioxidant activity of fermented whole-plant cassava surpassed that of anaerobically fermented mulberry leaf and alfalfa, possibly due to inherent differences in the antioxidant capacity of the raw materials themselves [28,42]. Ekeledo et al. [43] also reported cassava’s excellent antioxidant potential, consistent with our research findings. Additionally, the literature suggests that PS contains higher amounts of active constituents, such as phenolics and flavonols, which may enhance oxidation activity [17,18,34]. The increased oxidation activity observed in PS-containing fermentation is likely attributable to the higher concentration of these active constituents, acting as antioxidants. The higher concentration of active constituents during fermentation likely enhances the scavenging of O_2_, further bolstering antioxidant capacity [3,28]. Furthermore, the lower pH in PS-containing fermentation may restrict the synthesis and assembly of CAT protein, a inhibiting CAT activity, and thereby augmenting antioxidant capacity [28,42]. Overall, anaerobic fermentation with the addition of PS proves to be advantageous in antioxidant capacity. The body’s antioxidant capacity is closely related to health, the impact of environmental biotic or abiotic stress on humans is becoming increasingly severe. The demand for food to combat oxidative stress is very urgent, and there is great potential to produce functional foods by utilizing the strong antioxidant ability of fermented PS.

### 3.3. Bacterial Community Structure and Predicted Functions in Co-Fermented Whole-Plant Cassava and PS

**Figure 1:**
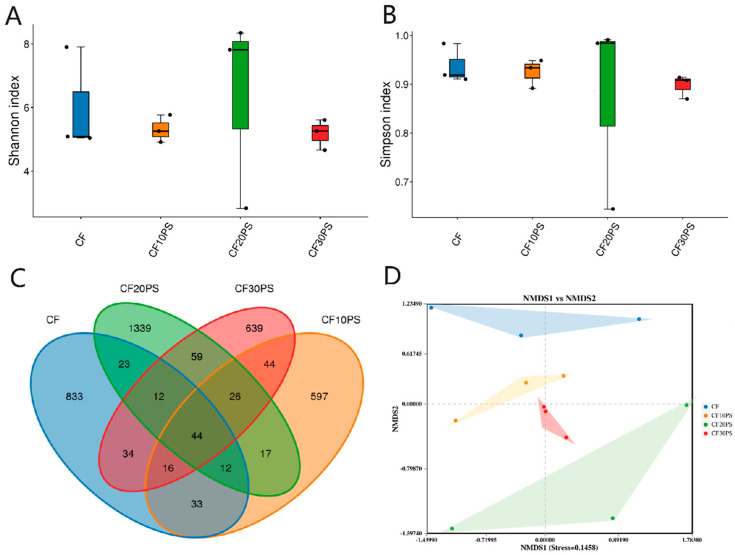
The bacterial diversity of co-fermented whole-plant cassava and PS. (**A**) The Shannon index and (**B**) the Simpson index of the bacterial communities in the co-fermented whole-plant cassava and PS. (**C**) Venn diagram of the bacterial OTUs. (**D**) Non-metric multidimensional scaling (NMDS) analysis of the bacterial communities in the co-fermented whole-plant cassava and PS. CFCK: 100% whole-plant cassava without PS; CF10PS: 90% whole-plant cassava with 10% PS; CF20PS: 80% whole-plant cassava with 20% PS; CF30PS: 70% whole-plant cassava with 30% PS.

The results of α-diversity and β-diversity analyses of co-fermented cassava bacterial communities are shown in Figure 1. According to the lower Shannon and Simpson diversity index in the co-fermented treatments (except CF20PS), PS addition led to a decrease in α-diversity (Figure 1A,B). A total of 3728 OTUs were identified, with 44 shared OTUs across the four microbiomes. Among the treatment, the CF10PS treatment had the lowest unique OUT number (789), while the CF20PS treatment exhibited the highest (1532) (Figure 1C). In the NMDS analysis, the fermented cassava microbial communities varied significantly among the four treatments (Figure 1D), indicating a shift in response to the mixed ratio of whole-plant cassava and PS.

**Figure 2:**
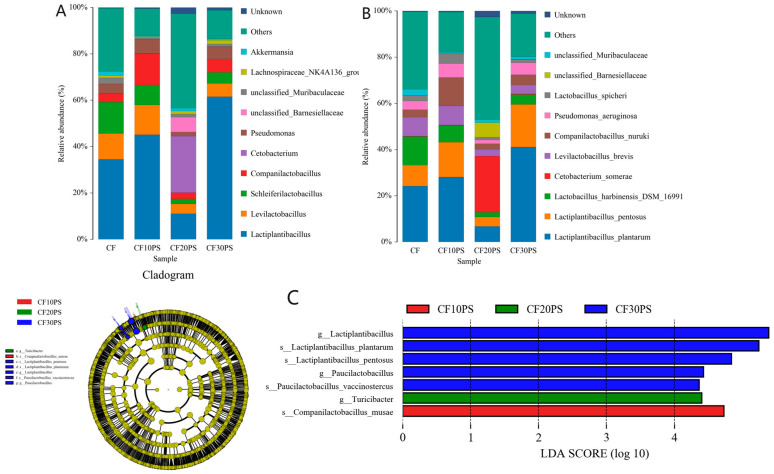
Relative abundance of bacteria at (**A**) genus and (**B**) species level in the co-fermented whole-plant cassava and PS. (**C**) Comparison of microbial variations and identification of indicator bacteria using LEfSe. CFCK: 100% whole-plant cassava without PS; CF10PS: 90% whole-plant cassava with 10% PS; CF20PS: 80% whole-plant cassava with 20% PS; CF30PS: 70% whole-plant cassava with 30% PS.

The composition of bacterial communities in co-fermented whole-plant cassava was further explored among the treatments (Figure 2A). In the CFCK treatment, *Lactiplantibacillus* (34.55%), *Schleiferilactobacillus* (13.56%), *Levilactobacillus* (12.20%), and *Pseudomonas* (4.06%) were the predominant genera identified. *Lactiplantibacillus* was the most abundant bacterial genus in co-fermented treatment, and significantly increased in the three mixed ferment treatments, other than in CF20PS (*p* < 0.05). Conversely, the undesirable *Cetobacterium* was markedly higher in the CF20PS treatment (*p* < 0.05). Within the co-fermented whole-plant cassava treatment, but excluding CF20SP, *Lactiplantibacillus*, *Levilactobacillus*, and *Companilactobacillus* were prominent genera, with their abundance varying as the proportion of PS increased. At the species level, *Lactiplantibacillus plantarum* and *Lactiplantibacillus pentosus* were abundant bacteria across all treatments. In the CF20PS treatment, the undesirable bacterial species *Cetobacterium somerae* had the highest abundance (Figure 2B). Notably, the trends in bacterial community composition at the genus level were consistent with those observed at the species level across all treatments.

We utilized the LEfSe method to discern differences in bacterial communities within co-fermented whole-plant cassava across various treatments, leading to the identification of specific microbial taxa unique to each treatment (Figure 2C). Some indicator bacteria were enriched in all three treatments. In the CF10PS treatment, *Companilactobacillus musae* was the indicator bacteria, and *Turicibacter* genera was enriched in the CF20PS treatment; while lactic acid bacteria genera, including *Lactiplantibacillus* and *Paucilactobacillus*, as well as specific species like *Lactiplantibacillus plantarum*, *Lactiplantibacillus pentosus*, and *Paucilactobacillus vaccinostercus*, were more abundant in the CF30PS treatment.

**Figure 3:**
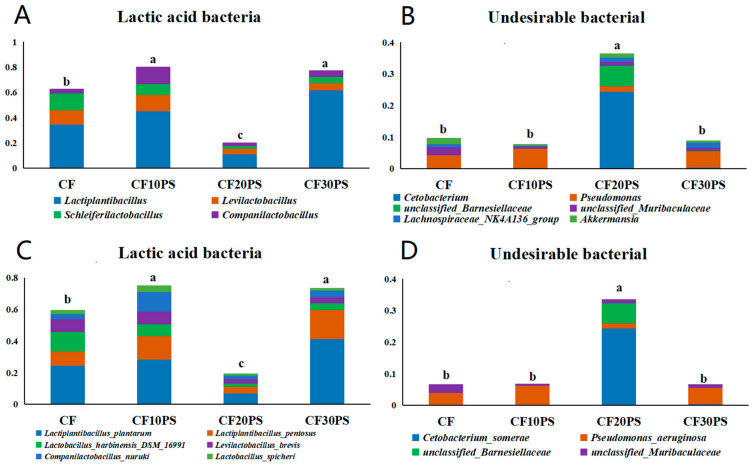
Abundance of lactic acid bacteria at the (**A**) genus and (**C**) species level in the co-fermented whole-plant cassava and PS. Abundance of undesirable bacteria at the (**B**) genus and (**D**) species level in the co-fermented whole-plant cassava and PS. CFCK: 100% whole-plant cassava without PS; CF10PS: 90% whole-plant cassava with 10% PS; CF20PS: 80% whole-plant cassava with 20% PS; CF30PS: 70% whole-plant cassava with 30% PS. The different letters in figures are significantly different (*p* < 0.05).

To understand the dynamics of microorganisms crucial for anaerobic fermentation, we assessed the total abundance of lactic acid bacteria and undesirable bacteria among different treatments (Figure 3). Lactic acid bacteria included genera such as *Lactiplantibacillus*, *Levilactobacillus*, *Schleiferilactobacillus*, and *Companilactobacillus*, along with their corresponding species-level microorganisms (Figure 3A,C). Meanwhile, undesirable bacteria included *Cetobacterium*, *Acetobacter*, *Pseudomonas*, unclassified_*Barnesiellaceae*, unclassified_*Muribaculaceae, Lachnospiraceae_NK4A136_group*, and *Akkermansia* genera, and species (Figure 3B,D). Comparing co-fermentation treatments with individual whole-plant cassava fermentation, there was a trend of increasing lactic acid bacteria in the co-fermentation treatments, except for CF20PS, although these treatments did not significantly reduce reducing undesirable bacteria. Notably, the CF20PS treatment exhibited the lowest lactic acid bacteria and the highest undesirable bacteria abundance (*p* < 0.05). This outcome aligns with the variation in fermentation quality observed earlier in the CF10PS and CF20PS treatments, with CF10PS showing the best quality and CF20PS exhibiting the poorest quality among the treatments.

Typically, as the anaerobic fermentation process advances, LAB tend to dominate, simplifying the bacterial community structure and thereby reducing alpha diversity in well-preserved products [23,25]. However, our study reveals a higher alpha diversity in the CF20PS group, which has the poorest fermentation quality. This could be attributed to the presence of more undesirable bacteria (such as *Cetobacterium*, *Acetobacter*, *Pseudomonas*, unclassified_*Barnesiellaceae*, unclassified_*Muribaculaceae*, *Lachnospiraceae_NK4A136_group*, and *Akkermansia*) in the CF20PS group, enhancing biodiversity. Dike et al. [44] conducted bacterial 16S rRNA sequencing (targeting the V3–V4 region) of fermented cassava food, noting a relatively low diversity index compared to our study. The disparity may be due to variations in raw materials and sequencing methods and that their study focuses on cassava tuber, which is primarily made up of carbohydrates (>80%). In our research, whole-plant cassava, rich in nutritional components such as CP, fiber, WSC, starch, and minerals, served as a fermentation substrate, fostering the proliferation and growth of a diverse microbial population. Furthermore, our study employed third-generation sequencing of the full-length 16S rRNA (targeting the V1–V9 region), providing a wider sequencing area, greater depth, and accuracy in species identification compared to next-generation sequencing. This technology’s advantages have been validated in other anaerobic ferment studies [27]. Additionally, our research highlights the significant impact of co-fermentation treatments on β-diversity. Bacterial communities distinctly separated in different ferment groups, a phenomenon consistent with other co-fermentation studies [29,39].

In the present study, we detailed the bacterial community composition of fermented whole-plant cassava at the species taxonomic level, revealing *Lactiplantibacillus plantarum, Lactiplantibacillus pentosus*, and *Lactobacillus harbinensis* as the dominant bacteria. This dominance indicated a primarily lactic acid fermentation mode, ensuring high fermentation quality. Surprisingly, there is limited literature exploring the bacterial community structure of fermented cassava products. Adesulu-Dahunsi et al. (2022) [13] emphasized the significant role of *Lactobacillus* in African indigenous fermented foods, mainly cassava-based. In another study by Dike et al. [44], *Lactococcus* and *Pseudomonas* were found to be the predominant bacteria in fermented cassava tuber food. *Lactococcus*, belonging to LAB, plays a pivotal role in anaerobic fermentation [24,25]. *Pseudomonas* may be associated with protein degradation and is generally considered unfavorable for anaerobic fermentation [45]. Notably, *Pseudomonas*, *Bacillus*, *Paenibacillus*, and *Clostridium* were dominant microbes in fermented cassava leaves, indicating low fermentation quality [38,46]. These findings are consistent with our study, where a lower abundance of *Pseudomonas* was observed in all fermented whole-plant cassava groups, although its precise function remains unclear.

Furthermore, the co-fermentation of whole-plant cassava and PS led to an optimized silage microbial community structure, which increased the abundance of lactic acid bacteria (up to 80%), except for the CF20PS treatment. Similar dynamic trends have been observed in previous studies. Wang et al. [24,39] reported a remarkable increase in LAB abundance (*L. buchneri*, *L. hilgardii*, or *L. plantarum*) and a reduction to zero in undesirable *Bacillus* and *Enterococcus* after co-fermenting different raw materials. Another study found that *Lactobacillus* (approximate 70%) dominated in a mixed fermentation product of soybean and corn [29]. Interestingly, in our study, we discovered more dominant bacteria in well-preserved fermented groups, such as *Lactiplantibacillus plantarum*, *Lactiplantibacillus pentosus*, *Lactobacillus harbinensis*, *Levilactobacillus brevis*, *Companilactobacillus nuruki*, and *Lactobacillus spicheri.* This finding deviates significantly from previous research results, warranting further investigation to explain this phenomenon.

**Figure 4:**
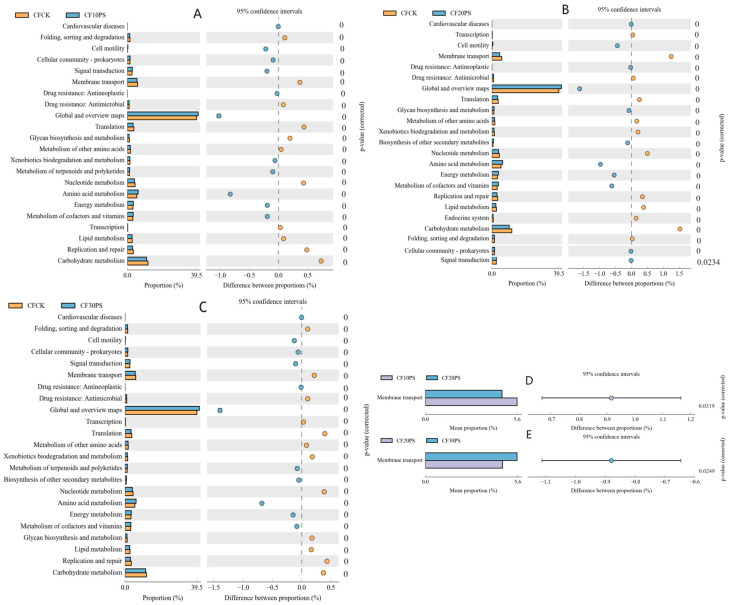
Comparison of microbial function pathways: Detected pathway enrichment of (**A**) CFCK vs. CF10PS, (**B**) CFCK vs. CF20PS, (**C**) CFCK vs. CF30PS, (**D**) CF10PS vs. CF20PS, and (**E**) CF20PS vs. CF30PS. CFCK: 100% whole-plant cassava without PS; CF10PS: 90% whole-plant cassava with 10% PS; CF20PS: 80% whole-plant cassava with 20% PS; CF30PS: 70% whole-plant cassava with 30% PS.

Co-fermentation of whole-plant cassava and PS significantly altered the microbial community structure compared with individual fermentation of whole-plant cassava. This resulted in substantial diversity in predicted metabolic functions, as revealed through analysis using PICRUSt2 software (v2.3.0_b) (Figure 4). When comparing CFCK to CF10PS, CF20PS, and CF30PS, similar significant differences were observed in their KEGG pathways (Figure 4A–C). The CFCK treatment exhibited higher enrichment in up-regulation pathways such as carbohydrate metabolism, membrane transport, nucleotide metabolism, lipid metabolism, replication and repair, and translation. Conversely, the three co-fermentation treatments showed up-regulation pathways in global and overview maps, amino acid metabolism, energy metabolism, metabolism of cofactors and vitamins, and signal transduction. Additionally, the KEGG pathways of membrane transport was significantly down-regulated in the CF20PS treatment group compared with the CF10PS and CF30PS treatments (*p* < 0.05) (Figure 4D,E). Overall, the impact of co-fermentation on microbial prediction function was significantly greater than that of whole-plant cassava fermentation alone, particularly concerning the metabolism of nutrients, genetic information processing, and environmental information processing.

Anaerobic fermentation, a complex micro-ecological process, is shaped by diverse microbial communities that influence metabolic products, thereby regulating fermentation quality. The predictive function of the microbial community offers valuable insight into their role within fermentation systems [47]. In our study, the most prevalent pathway identified was global and overview maps, which was significantly up-regulated in the co-fermentation groups. This highlights the pivotal role of this pathway in microbial metabolism and aligns with previous findings reported by Du et al. [48]. Carbohydrate metabolism, which is linked to lactic acid bacteria abundance in silage microbial communities, was notably influenced in the co-fermentation treatments. These treatment groups exhibited a higher abundance of lactic acid bacteria, consequently leading to an up-regulation in carbohydrate metabolism. This observation echoes results reported by Wang et al. [24] and Li et al. [15], indicating a consistent trend.

Amino acid metabolism emerged as another vital pathway that is related to nitrogen and amino acid degradation. The co-fermentation treatments exhibited lower crude protein (CP) and ammonia-N content, coupled with a remarkable up-regulation of the amino acid metabolism pathway. This finding concurs with similar results reported by Bai et al. [27] and Li et al. [15], underscoring the importance of this pathway in regulating fermentation outcomes. Additionally, pathways related to genetic functions and environmental adaptation were enriched in the co-fermentation treatments groups. This enrichment may be attributed to the adaptive responses of microbial communities to acidic environments during fermentation [27]. Consequently, the diverse microorganisms in the fermentation system contribute to varied metabolic pathways, thereby finely tuning and regulating the quality of fermentation outcomes. However, in this study, the predictive function of the microbial community is based solely on 16s sequencing results, due to limitations in sequencing depth and databases, and the accuracy of functional prediction results is low, making it impossible to perform functional analysis of special functional genes, such as antibiotic resistance genes (ARGs) and carbohydrate active enzymes (CAZy). Therefore, it is necessary to use metagenomic technology to analyze the gene function of ferment food microorganisms.

### 3.4. Bacterial Co-Occurrence Network, Network Modules, and Stability in Co-Fermented Whole-Plant Cassava and PS

**Figure 5:**
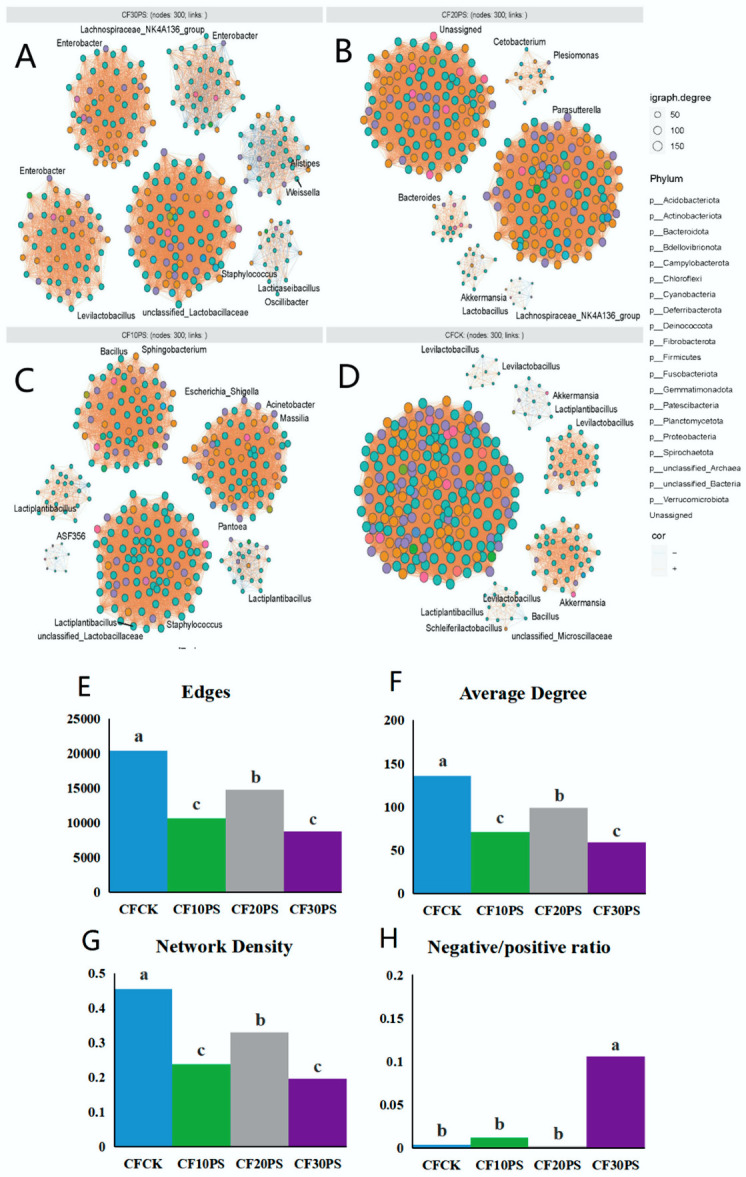
The bacterial community co-occurrence networks of the co-fermented whole-plant cassava and PS. (**A**) CF30PS, (**B**) CF20PS, (**C**) CF10PS, and (**D**) CFCK, (**E**) numbers of edges, (**F**) average degree, (**G**) network density, and (**H**) negative/positive ratios of the bacterial community co-occurrence networks. CFCK: 100% whole-plant cassava without PS; CF10PS: 90% whole-plant cassava with 10% PS; CF20PS: 80% whole-plant cassava with 20% PS; CF30PS: 70% whole-plant cassava with 30% PS. The different letters in figures are significantly different (*p* < 0.05).

The co-occurrence networks of co-fermented whole-plant cassava and PS reveals distinct patterns (Figure 5A–D). In the CFCK group, the network exhibited the highest number of networks, network density, and average degrees (Figure 5E–G), signifying more intricate microbial community structures and interactions compared to co-fermentation treatment groups. The stability of microbial networks was evaluated based on the ratio of negative to positive interactions, with higher ratios indicating superior network stability. Remarkably, the CF30PS treatment group displayed the significantly highest negative/positive interaction ratio (*p* < 0.05), indicating a heightened stability in bacterial networks.

**Figure 6:**
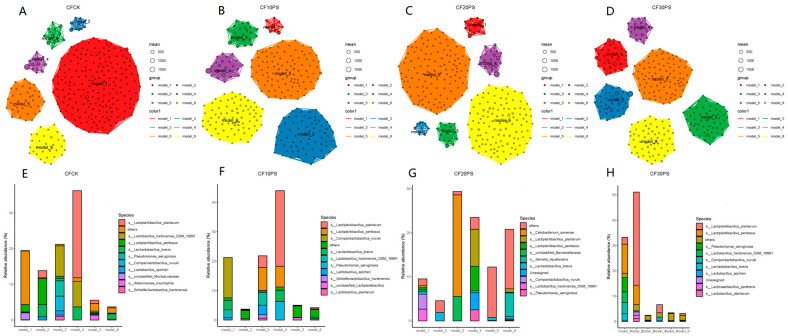
Modules and composition in the bacterial community co-occurrence networks. CFCK: 100% whole-plant cassava without PS (**A**,**E**); CF10PS: 90% whole-plant cassava with 10% PS (**B**,**F**); CF20PS: 80% whole-plant cassava with 20% PS (**C**,**G**); CF30PS: 70% whole-plant cassava with 30% PS (**D**,**H**).

The co-occurrence network modules for co-fermented whole-plant cassava and PS are depicted in Figure 6. In the CFCK silage group, the network comprised six modules, with module 4 being predominant. Abundant bacteria in this module included *Lactiplantibacillus plantarum*, *Lactobacillus harbinensis*, and *Levilactobacillus brevis.* Undesirable bacteria, including unclassified_*Muribaculaceae*, *Pseudomonas aeruginosa* and *Akkermansia muciniphila*, were found in lower abundance in modules 1, 3, and 5. Similar composition of dominant modules was observed in the CF10PS and CF30PS treatments, where *Lactiplantibacillus plantarum* and *Lactiplantibacillus plantarum* were the top two species in terms of richness.

Notably, in the CF20PS treatment group, *Cetobacterium somerae* was the major bacterium in module 3 of the bacterial network, indicating its dominance. Consequently, this group exhibited lower lactic acid bacteria abundance and higher undesirable bacteria compared to other co-fermentation groups, suggesting that the dominant presence of *Cetobacterium somerae* in module 3 might influence the bacterial community. Overall, co-fermentation treatments significantly altered the numbers, pattern size, distributions, and relative abundances of these modules in the bacterial network. The ratio of PS added proved to be a crucial factor contributing to the diversity observed in microbial network modules.

**Figure 7:**
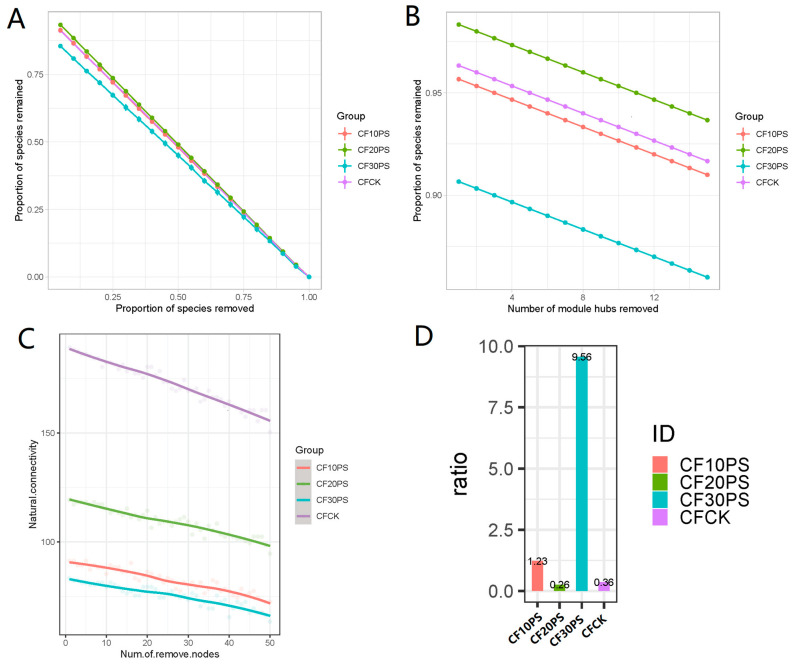
Robustness of microbial networks (**A**) random and (**B**) targeted removal of module hubs. (**C**) Natural connectivity and (**D**) negative correlation ratio of the microbial networks. CFCK: 100% whole-plant cassava without PS; CF10PS: 90% whole-plant cassava with 10% PS; CF20PS: 80% whole-plant cassava with 20% PS; CF30PS: 70% whole-plant cassava with 30% PS.

The diversity of microbial network modules can significantly impact the functionality and stability of a fermentation micro-ecosystem. Consequently, we calculated multiple stability indices—robustness, natural connectivity, and negative correlation ratio—to elucidate the effects of PS added ratios on the microbial network stability of co-fermented whole-plant cassava and PS (Figure 7). Notably, the CF30PS treatment group exhibited considerably higher robustness compared to the other treatment groups (Figure 7A,B). Additionally, the natural connectivity of bacterial networks decreased in the CF30PS group compared to CFCK and other co-fermented groups, indicating a stronger resistance in CF30PS (Figure 7C). Moreover, the negative correlation ratio of CF30PS silage group was remarkable higher than the other treatment groups (Figure 7D). Furthermore, the CF10PS treatment group demonstrated higher network stability compared to the CFCK and CF20PS groups. Overall, these results suggest that the co-fermentation of whole-plant cassava and PS enhances the stability of the silage microbial network compared to whole-plant cassava fermented alone. This enhancement could be attributed to the increased network complexity associated with connectivity and relative modularity. Notably, even small changes in PS addition had a remarkable impact on network stability.

**Figure 8:**
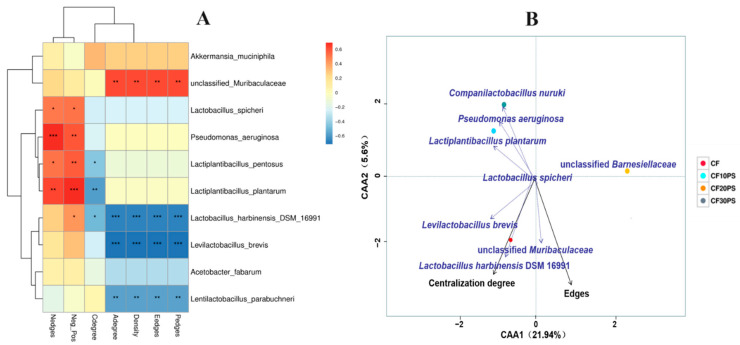
The correlation analysis between the network properties and microbial community of co-fermented whole-plant cassava and PS. (**A**) Correlation heatmap of network properties and microbial community. Positive and negative correlations are shown in red and blue, respectively. The color intensity is proportional to the correlation values. * *p* < 0.05, ** *p* < 0.01, *** *p* < 0.001. (**B**) Canonical correlation analysis of network properties and microbial community.

Furthermore, to investigate the impact of microbial community composition on the microbial networks, we conducted correlation heatmaps and canonical correlation analysis (CCA) between network properties and the microbial community of co-fermented whole-plant cassava and PS (Figure 8). The correlation heatmap reveals significant positive correlations between negative edges, negative/positive ratio, and *Lactiplantibacillus plantarum*, *Lactiplantibacillus pentosus*, and *Pseudomonas aeruginosa* (*p* < 0.05). Additionally, edges, positive edges, edge density, and average degree exhibited negative correlations with *Lactiplantibacillus plantarum* and *Lactiplantibacillus pentosus* (*p* < 0.05) (Figure 8A). The canonical correlation analysis (Figure 8B) indicates that centralization degree and edges are notably influenced by microbial community composition, with similar impact degrees. Centralization degree was positively influenced by *Lactobacillus harbinensis*_DSM_16991 and *Levilactobacillus brevis.* Edges were largely positive impacted by unclassified_*Muribaculaceae*. These result underscore the critical role of key bacterial species in shaping characteristics of microbial networks.

From an ecological perspective, each individual anaerobic fermentation package represents a microbial ecosystem [24,47]. The existing literature suggests that changes in the ecosystems network structure can affect their functionality and stability at a macroscopic scale. The relationships between network complexity and stability has been explored to some extent [49,50]. Yuan et al. [20] supported these findings by analyzing the complexity and stability of molecular ecological networks in soil microbial communities. In the past two years, researchers have focused on analyzing microbial community networks. Various factors such as environmental conditions, modulation methods, fermentation duration, and silage materials significantly impact microbial networks. Dong et al. [25] demonstrated that the epiphytic microbiota of raw materials harvested at different times of the day can influence the complexity of the ferment bacterial networks, with morning-harvested raw material exhibiting greater stability. Bai et al. [23] explored the bacterial network properties of fermented corn and found that storage temperature has a substantial impact on network complexity, surpassing the influence of LAB additive treatments. Moreover, Wang et al. [24] reported increased bacterial network stability in co-fermented products as fermentation progresses. Recently, we reported the effect of whole plant cassava mixed with another feed corn silage on microbial networks, and the addition of corn reduced the stability of the network [22]. However, the effects of whole-plant cassava co-fermentation with medicinal plants on bacterial network modules, complexity, and stability remain largely unknown. Our results suggest that co-fermenting whole-plant cassava and PS changes the stability of the silage microbial network compared to whole-plant cassava fermented alone. This improvement can be attributed to altered network complexity associated with connectivity and relative modularity. Importantly, even small changes in PS addition significantly impact network stability. Further, we observed a strong interaction between dominant bacterial species and microbial network characteristics. Network complexity was influenced by dominant LAB, while stability was largely affected by undesirable bacteria, such as *Pseudomonas aeruginosa* and unclassified_*Muribaculaceae*. Therefore, we believe that PS has a positive impact on the complexity and stability of bacterial networks. Lower abundances of undesirable bacteria play a crucial role in the complexity and stability of microbial ecological networks.

However, in the present study, the changes in medicinal components before and after whole-plant cassava and PS co-fermentation were not involved, and the role of the functional components in antioxidant ability, microbial communities, and microecological networks of ferment food have not yet been elucidated. These will be possible areas of future research, and shown great significance for the development and production of high-quality functional food of whole-plant cassava and PS.

## 4. Conclusions

The co-fermentation of whole-plant cassava with 10% PS resulted in excellent fermentation quality and enhanced antioxidant ability, making it a promising approach for functional food production. PS addition not only increased the total abundance of lactic acid bacteria, but also influenced the predicted functions of the bacterial community when compared to whole-plant cassava fermented alone. Furthermore, our analysis of co-occurrence networks reveals that co-fermentation impacts microbial network features, including module numbers and bacterial relative abundances, leading to altered complexity and stability of the networks. It was evident that microbial community composition played a pivotal role in shaping these network properties, with undesirable species like *Pseudomonas aeruginosa* and unclassified_*Muribaculaceae* significantly influencing network complexity and stability. However, the role of the functional components of PS in microbial communities and microecological networks has not yet been elucidated, which may be a direction for future research. In summary, this study provided valuable insights into the microbial community and microecological network of co-fermentation, and sheds light on the microbial mechanisms underpinning the production of high-quality functional foods through co-fermentation with medicinal plants.

## Figures and Tables

**Table 1:** Chemical composition and epiphytic microbial counts of fresh whole-plant cassava and *Piper sarmentosum*.

Items	Abbreviation	Whole-Plant Cassava	*Piper sarmentosum*	SEM	*p*-Value
Dry matter (g/kg FM)	DM	312.5 ± 17.18 a	192.4 ± 15.34 b		<0.05
Crude protein (g/kg DM)	CP	158.6 ± 9.64 a	41.9 ± 2.55 b		<0.05
Acid detergent fiber (g/kg DM)	ADF	116.8 ± 8.73 a	62.5 ± 4.38 b		<0.05
Neutral detergent fiber (g/kg DM)	NDF	158.9 ± 16.41 a	86.1 ± 6.04 b		<0.05
Water soluble carbohydrates (g/kg DM)	WSC	177.2 ± 22.5 a	38.7 ± 3.59 b		<0.05
Starch (g/kg DM)		215.9 ± 14.6 a	12.4 ± 0.83 b		<0.05
Lactic acid bacteria (Log cfu/g FM)	LAB	5.76 ± 0.45	5.28 ± 0.66		>0.05
Mold (Log cfu/g FM)		2.43 ± 0.18 a	1.52 ± 0.12 b		<0.05
*Enterobacter* (Log cfu/g FM)		2.68 ± 0.23 a	1.73 ± 0.17 b		<0.05

Note: SEM, standard error of the mean. Means within the same row with different letters are significantly different (*p* < 0.05).

**Table 2:** Nutrition composition, fermentation characteristics, and antioxidant capacity of co-fermented whole-plant cassava and PS.

Items	Treatments				SEM	*p*-Value	
	CFCK	CF10PS	CF20PS	CF30PS		T	L
Nutrition composition, g/kgDM							
DM (g/kg FM)	301.3 ± 20.7 a	294.0 ± 15.6 b	283.5 ± 17.6 c	269.2 ± 18.1 d	7.0	<0.05	<0.05
CP (DM)	142.5 ± 7.1 a	131.8 ± 8.0 b	121.6 ± 9.6 c	111.7 ± 7.9 d	6.6	<0.05	<0.05
ADF	94.1 ± 6.2 a	89.3 ± 4.3 a	85.7 ± 8.3 b	81.8 ± 3.9 b	2.6	<0.05	<0.05
NDF	132.4 ± 10.7 a	125.8 ± 7.9 ab	120.6 ± 11.4 b	115.4 ± 6.3 c	3.6	<0.05	<0.05
WSC	92.0 ± 6.6 a	84.8 ± 3.4 b	76.5 ± 7.0 c	70.4 ± 4.5 d	4.7	<0.05	<0.05
Starch	155.4 ± 14.8 a	138.6 ± 9.2 b	125.2 ± 13.5 c	109.7 ± 7.9 d	9.7	<0.05	<0.05
Fermentation characteristics, g/kg DM							
pH	4.27 ± 0.1 a	4.08 ± 0.07 b	4.33 ± 0.12 a	4.03 ± 0.06 b	0.1	<0.05	>0.05
Lactic acid	46.93 ± 4.5 b	58.24 ± 2.7 a	40.7 ± 3.6 c	61.8 ± 3.1 a	2.8	<0.05	>0.05
Acetic acid	19.28 ± 1.5 b	11.2 ± 0.8 c	30.5 ± 2.5 a	9.7 ± 0.7 c	5.8	<0.05	>0.05
Propionic acid	4.16 ± 0.12 a	1.5 ± 0.05 b	1.8 ± 0.06 b	1.6 ± 0.05 b	0.1	<0.05	>0.05
Butyric acid	0.88 ± 0.07 a	N	N	N	N	<0.05	>0.05
NH_3_-N/Total N	85.78 ± 6.1 a	45.4 ± 2.7 c	61.2 ± 3.2 b	47.3 ± 2.8 c	4.3	<0.05	>0.05
Antioxidant capacity, U/g FM							
T-AOC	112 ± 16 b	267 ± 20 a	283 ± 19 a	274 ± 17 a	40.8	<0.05	>0.05
SOD	347 ± 25 b	725 ± 46 a	740 ± 33 a	751 ± 50 a	98.1	<0.05	>0.05
GSH-Px	408 ± 28 b	954 ± 38 a	893 ± 44 a	931 ± 52 a	130.1	<0.05	>0.05
CAT	15.6 ± 1.3 a	8.3 ± 0.8 b	7.9 ± 0.5 b	8.6 ± 0.6 b	1.8 b	<0.05	>0.05

Note: CFCK: 100% whole-plant cassava without PS; CF10PS: 90% whole-plant cassava with 10% PS; CF20PS: 80% whole-plant cassava with 20% PS; CF30PS: 70% whole-plant cassava with 30% PS.T-AOC, total antioxidant capacity; SOD, superoxide dismutase; GSH-Px, glutathione peroxidase; CAT, catalase; T, treatment; L, linear; SEM, standard error of the mean. Means within the same row with different letters are significantly different (*p* < 0.05).

## Data Availability

The original contributions presented in the study are included in the article, further inquiries can be directed to the corresponding author.
